# Look Beyond Additivity and Extensivity of Entropy for Black Hole and Cosmological Horizons

**DOI:** 10.3390/e26100814

**Published:** 2024-09-25

**Authors:** Mariusz P. Da̧browski

**Affiliations:** 1 Institute of Physics, University of Szczecin, Wielkopolska 15, 70-451 Szczecin, Poland; mariusz.dabrowski@usz.edu.pl; 2 National Centre for Nuclear Research, Andrzeja Sołtana 7, 05-400 Otwock, Poland; 3 Copernicus Center for Interdisciplinary Studies, Szczepańska 1/5, 31-011 Kraków, Poland

**Keywords:** thermodynamics, entropy, additivity, extensivity, long-range interactions, horizons

## Abstract

We present a comparative analysis of the plethora of nonextensive and/or nonadditive entropies which go beyond the standard Boltzmann–Gibbs formulation. After defining the basic notions of additivity, extensivity, and composability, we discuss the properties of these entropies and their mutual relations, if they exist. The results are presented in two informative tables that are of strong interest to the gravity and cosmology community in the context of the recently intensively explored horizon entropies for black hole and cosmological models. Gravitational systems admit long-range interactions, which usually lead to a break of the standard additivity rule for thermodynamic systems composed of subsystems in Boltzmann–Gibbs thermodynamics. The features of additivity, extensivity, and composability are listed systematically. A brief discussion on the validity of the notion of equilibrium temperature for nonextensive systems is also presented.

## 1. Introduction

It is widely known that Boltzmann–Gibbs thermodynamics (from now on BG) and statistical mechanics are additive and extensive [1]. The core physical quantity that refers to these theories is entropy, which is assumed to be extensive, since it relates to the negligence of the long-range forces between thermodynamic subsystems. This assumption is justified only when the size of the system exceeds the range of the interactions between its components. As a result, the total entropy of a composite system is equal to the sum of the entropies of the individual subsystems (additivity), and the entropy grows with the size of the system or its configuration space (extensivity).

However, contemporary physics exhibits a number of systems for which the long-range forces are important. Examples of such systems are gravitational systems, since gravity is long-range and interactive, and it is strongly non-linear when its extreme regimes are taken into account. Strong gravity characterizes all of the compact astrophysical objects in the universe, like white dwarfs, neutron and boson stars, quark stars, etc., with the most extreme and most intriguing being black holes. The latter are surrounded by horizons with areas that, according to Bekenstein and Hawking [2,3], can be interpreted as entropy. Therefore, it is possible to formulate the appropriate laws of thermodynamics. Since for black holes, the Bekenstein entropy *scales with the area* and not with the volume (size), it is consequently a nonextensive quantity [4,5,6,7,8,9]. Additionally, because of the long-range interaction nature of gravity, Bekenstein entropy is also nonadditive.

In fact, a number of nonadditive and/or nonextensive entropies have been proposed in the literature [10,11,12,13,14,15,16]. Most of them have been applied to gravitational systems both in astrophysics and in cosmology, and there is a debate regarding whether they can serve dark energy [17,18,19,20,21,22,23,24,25,26,27,28,29,30,31,32,33,34], which is specifically called *holographic dark energy* [35]. Amazingly, a number of these explorations do not acknowledge the nonextensivity of these systems, which creates some issues related to their firm thermodynamical background [36]. This motivates us to investigate the problem of nonextensive entropy applications in a gravitational framework.

In this paper, we explore the topics of additivity and extensivity of entropies, which go beyond standard BG thermodynamics, being strongly motivated by gravitational interactions. Our focus is on non-standard entropies that are better fitting to gravitational systems, such as Bekenstein entropy [2,3], Tsallis *q*-entropy [10,37], Tsallis–Cirto δ-entropy [4], Barrow Δ-entropy [16], Tsallis q,δ entropy, Tsallis–Jensen q,γ-entropy [38], Rényi entropy [11], Landsberg *U*-entropy [39], Sharma–Mittal entropy [12,13], and Kaniadakis entropy [14,15].

The following is the outline of this paper. In Section 2, we define additivity and extensivity in thermodynamic systems and try to establish some generalities about possible composition rules for entropy. In Section 3, we go beyond the definitions of additivity and extensivity. In Section 4, we constructively review and compare the plethora of nonadditive and/or nonextensive entropies, together with accompanying nonadditive and/or nonextensive thermodynamical quantities. Then, we classify the entropies under study with respect to their additivity and extensivity properties, as well as with the application of the appropriate composition rules. Finally, in Section 5, we summarize this paper.

## 2. Boltzmann–Gibbs Thermodynamics and Statistical Mechanics

Boltzmann–Gibbs thermodynamics and statistical mechanics are based on two key hypotheses: that the entropy is extensive, and that the internal energy and entropy follow the additive composition rule for a system made of some subsystems. All physical relations in BG statistical mechanics are defined in light of these conditions, which, in fact, rely on ignoring long-range forces between thermodynamic subsystems.

Boltzmann–Gibbs (BG) entropy is defined as follows [1]:

(1) $$S_{BG}=-k_{B}\sum_{i=1}^{n}p_{i}\ln p_{i}=k_{B}\sum_{i=1}^{n}p_{i}\ln\frac{1}{p_{i}},$$

where $p_{i}$ is the probability distribution defined in a configuration space Ω. The number of degrees of freedom (states) is *n*, $k_{B}$ is the Boltzmann constant, and the condition that the total probability must be equal to one $\sum p_{i}=1$ is fulfilled. For the case of all probabilities being equal, i.e., for $p_{i}=$ const. =p, we obtain the following:

(2) $$\sum_{i=1}^{n}p_{i}=1=np\;\Rightarrow p=1/n.$$

After applying (2) to (1), one obtains the following:

(3) $$S_{BG}=k_{B}\ln n,$$

which means that the entropy is proportional to the number of states *n* in the configuration space Ω.

In view of the key properties of BG thermodynamics, and in the context of our investigations beyond these properties, we can define additivity and extensivity in a general way based on the literature [39,40,41] as follows.

### 2.1. Additivity

Additivity means that for a given physical or thermodynamical quantity *f*, the following composition rule is fulfilled:

(4) $$f\left(A+B\right)=f\left(p_{A\cup B}\right)=f\left(p_{A}p_{B}\right)=f\left(p_{A}\right)+f\left(p_{B}\right)=f\left(A\right)+f\left(B\right),$$

where A and B are independent subsystems, each equipped with a set of configuration space degrees of freedom, $\Omega_{A}$ and $\Omega_{B}$, and corresponding probabilities, $p_{A}$ and $p_{B}$. The composite system A∪B has the probability $p_{A\cup B}$, and it is equipped with a set of configuration space degrees of freedom $\Omega_{A\cup B}$. If the subsystems *A* and *B* are assumed to be independent, then the probabilities are related by $p_{A\cup B}=p_{A}p_{B}$, which allows for the transition leading to the additivity rule (4) [42].

If a particular case of the entropy *S* is taken into account, then (4) reads as follows:

(5) S(A+B)=S(A)+S(B).

### 2.2. Extensivity

Let us assume that there is a set of physical quantities $\left(X_{0},X_{1},X_{2},\ldots ,X_{k}\right)$ such that $X_{0}=f\left(X_{1},X_{2},\ldots ,X_{k}\right)$. The extensivity of a selected physical quantity means that the function *f* that describes this quantity is *homogeneous degree one* [1,39,40]; i.e., that

(6) $$f\left(aX_{1},aX_{2},\ldots ,aX_{k}\right)=af\left(X_{1},X_{2},\ldots ,X_{k}\right)$$

for every positive real number a>0, for all $X_{1},X_{2},\cdots X_{k}$. Taking k=3, we have only four quantities, $X_{0},X_{1},X_{2},X_{3}$, and assuming that they are the entropy *S*, the energy *E*, the volume *V*, and the mole number *N*, accordingly, we can obtain the standard Boltzmann–Gibbs thermodynamic extensivity relation for the entropy [39]:

(7) S(aE,aV,aN)=aS(E,V,N).

In fact, the property (6) is called ’homogeneity’, and it is considered the most general definition of extensivity (cf. [39]).

In standard textbooks on thermodynamics, one commonly uses a less general definition of an extensive quantity, which says that if a system’s total number of states in the configuration space Ω is proportional to its number of degrees of freedom, then this quantity (such as the entropy, for example) is extensive. For BG entropy, as we have shown in (3), one has that $S_{BG}\left(n\right)=k_{B}\ln\left(n\right)\propto n$, where *n* is the total number of states in the system.

The advantage of definition (6) is that one does not refer to any kind of geometrical or bulk properties of a system such as the ’size’, though the geometrical size of a system seems intuitively to be related to the number of states or degrees of freedom.

### 2.3. Concavity

Concavity is the feature of the functions, which read as [4,39]

(8) f(ax+(1−a)y)≥af(x)+(1−a)f(y)(a>0).

In the context of thermodynamics, concavity of entropy guarantees that the system in thermodynamic equilibrium is *stable*.

## 3. Beyond Boltzmann–Gibbs Thermodynamics

### 3.1. Composability

Let us consider two independent systems, *A* and *B*, combined as a single Cartesian product A×B of the states of *A* and *B* with the requirement that [38]:

(9) $$S\left(A\times B,\Upsilon \right)=k_{B}g\left(\frac{S(A)}{k_{B}},\frac{S(B)}{k_{B}}\right),$$

where *g* is a smooth function of S(A), and S(B) and Υ is a parameter, which in the limit Υ→0, giving an additive composition rule (5). If the systems *A* and *B* fulfill the condition (9), then their combined system A+B is called *composable*. Of course, the BG system is composable.

### 3.2. Beyond Additivity

Additivity is violated if the rule (5) does not hold. In this case, one can have two options [39]. The first one is when

(10) S(A+B)≥S(A)+S(B),

which is called *superadditivity*, and it leads to the tendency of the system to clump its pieces/subsystems. The second one is when

(11) S(A+B)<S(A)+S(B),

which is *subadditivity*, and it tends to fragment the system into pieces rather than clumps. A cosmological similarity of such a system is a phantom [43,44], since it spontaneously splits into pieces under (anti)gravity, beginning with the largest-size pieces and terminating at the smallest [45].

In the literature, there are a number composition rules for nonadditive systems, which we introduce in Section 4. One of them, which generalizes the additive composition rule (5) into a nonadditive case, is the Abé rule [46,47,48]. It fulfills the composability requirement given by (9). If applied to entropy, it reads as follows

(12) $$S\left(A+B\right)=S\left(A\right)+S\left(B\right)+\frac{\Upsilon }{k_{B}}S\left(A\right)S\left(B\right),$$

where Υ takes numerical values according to the statistical definition of a specific entropy type. For BG entropy, one just has Υ=0. With the assumption that all the entropies in (12) are positive, one deals with superadditivity for Υ≥0, and with subadditivity for Υ<0. In fact, the physical interpretation of Υ is the result of the long-range interactions between subsystems, which leads to nonadditivity.

### 3.3. Beyond Extensivity

In BG thermodynamics, the additivity and the extensivity are closely related—additivity implies extensivity, and extensivity implies additivity [1]. This is not the case in general, and so the extensivity and the additivity may not be related, i.e., the extensivity may not imply the additivity, and vice versa. An example of such a kind of a quantity, which is based on the definition (6), is given by the function $f\left(X_{1},X_{2}\right)=x_{1}^{2}/\sqrt{X_{1}^{2}+X_{2}^{2}}$. It obeys extensivity, but not additivity [39].

Generally, the entropy *S* is nonextensive if

(13) S(aX)≠aS(X),

where *X* is a thermodynamical quantity and a>0, i.e., when the relations (6) and (7) are violated.

## 4. A Comparable Analysis of Nonextensive Entropy Plethora

### 4.1. Bekenstein Entropy

The Bekenstein entropy is not motivated by anything like statistical mechanics, but it is a well-established notion in gravity theory [2]. For a Schwarzschild black hole, it reads

(14) $$S_{Bek}=4\pi k_{B}{\left(\frac{M}{m_{p}}\right)}^{2}=\frac{4\pi k_{B}GM^{2}}{\hbar c},$$

and it is usually presented with its accompanying Hawking temperature, which reads

(15) $$T_{H}=\frac{\hbar c^{3}}{8\pi Gk_{B}M},$$

where *M* is the mass of a black hole, *c* is the speed of light, *G* is the gravitational constant, *ℏ* is the reduced Planck constant, and $m_{p}$ is the Planck mass. In fact, the temperature (15) can be calculated from the entropy (14) by applying the Clausius formula

(16) $$\frac{k_{B}}{T}=\frac{\partial S}{\partial E},$$

and using the Einstein mass–energy equivalence formula $E=Mc^{2}$.

It is not always understood in the literature that because of the area rather than volume scaling, the Bekenstein entropy is nonextensive, and that it obeys the following nonadditive composition rule (see e.g., [49])

(17) $$S_{A+B}=S_{A}+S_{B}+2\sqrt{S_{A}}\sqrt{S_{B}},$$

which we will call the *square root rule* from now on. This rule comes directly as a consequence of (14), according to which the entropy $S_{Bek}\propto M^{2}$, so that $S_{A}\propto M_{A}^{2}$, and $S_{B}\propto M_{B}^{2}$. If black holes merge in an adiabatic way, then their mass after merging is the sum $M_{A+B}=M_{A}+M_{B}$, but the entropy $S_{A+B}\propto M_{A+B}^{2}$, giving an extra term $1/2M_{A}M_{B}$, which is an extra nonadditive term in (17).

Curiously, after redefining the Bekenstein entropies as ${\tilde{S}}_{A+B}\equiv \sqrt{S_{A+B}}=M_{A+B}=M_{A}+M_{B}$, $\sqrt{S_{A}}=M_{A}\equiv {\tilde{S}}_{A}$, and $\sqrt{S_{B}}=M_{B}\equiv {\tilde{S}}_{B}$, one can rewrite the composition rule (17) in an additive way

(18) $${\tilde{S}}_{A+B}={\tilde{S}}_{A}+{\tilde{S}}_{B},$$

but this is not of any physical meaning.

In conclusion, the Bekenstein entropy addition formula (17) does not fulfill the Abé rule (12), though it looks quite similar. We comment on this point in relation to other entropies in Section 4.3.

### 4.2. Tsallis q, Tsallis–Cirto δ, Tsallis q,δ, and Tsallis–Jensen q,γ Entropies

#### 4.2.1. Tsallis *q*-Entropy

The Tsallis *q*-entropy [10,50] is one of the earliest proposals for the generalization of BG entropy. It encompasses an issue of the long-range interaction between thermodynamical subsystems by introducing a nonextensivity parameter *q* (q∈R) into the BG entropy definition (1), keeping the standard BG condition that the sum of all the probabilities is equal to one, i.e., that $\sum p_{i}=1$. It reads as follows:

(19) $$S_{q}=k_{B}\sum_{i=1}^{n}p_{i}\ln_{q}\frac{1}{p_{i}}=-k_{B}\sum_{i=1}^{n}{\left(p_{i}\right)}^{q}\ln_{q}p_{i}=-k_{B}\sum_{i=1}^{n}\ln_{2-q}p_{i},$$

where a newly defined *q*-logarithmic function, $\ln_{q}p$, is introduced:

(20) $$\ln_{q}p\equiv \frac{p^{1-q}-1}{1-q}.$$

In the limit q→1, one has the standard logarithm $\ln_{1}p=\ln p$. It is important that the *q*-logarithm does not fulfill the standard logarithm addition rule lnab=lna+lnb, where a,b are some arbitrary numbers. Instead, it fulfills a nonadditive composition rule given by

(21) $$\ln_{q}ab=\ln_{q}a+\ln_{q}b+\left(1-q\right)\ln_{q}a\ln_{q}b,$$

which, in fact, is the origin of the Abé rule (12). Interestingly, through the introduction of some specific q−product defined as [51]

(22) $${\left(x⊗y\right)}_{q}\equiv {\left[x^{1-q}+y^{1-q}-1\right]}^{\frac{1}{1-q}},\;\left(x\geq 0,y\geq 0\right),$$

one can make the rule (21) additive, i.e.,

(23) $$\ln_{q}\left[{\left(x⊗y\right)}_{q}\right]=ln_{q}x+\ln_{q}y.$$

It is also possible to define the *q*-exponential function

(24) $$e_{q}^{p}\equiv {\left[1+(1-q)p\right]}^{\frac{1}{1-q}},$$

which does not fulfill the standard exponent addition rule $e^{a+b}=e^{a}e^{b}$, though in the limit q→1, it does, since $e_{1}^{p}=e^{p}$.

The Tsallis *q*-entropy definition (19) is usually presented in three equivalent forms. However, using the definition of q-logarithm (20), all of them can be brought into the same form (cf. Appendix A):

(25) $$S_{q}=k_{B}\frac{1-\sum_{i=1}^{n}{\left(p_{i}\right)}^{q}}{q-1}.$$

It is important to mention that in order to fulfill the requirements of concavity for $S_{q}$ given by (8), the nonextensivity parameter should be positive q>0 [4].

In the limit q→1, the Tsallis entropy $S_{q}$ given by (19), or (25) reduces to BG entropy (1). After some check, it is possible to find that the Tsallis *q*-entropy (19) or (25) satisfies the nonadditive composition Abé rule (12) if one defines a nonextensivity parameter as Υ=1−q (cf. Appendix B). For equal probability states (2), the formula (25) gives the Tsallis *q*-entropy as

(26) $$S_{q}=k_{B}\ln_{q}n=k_{B}\frac{n^{q-1}-1}{1-q},$$

which nicely shows how it generalizes BG entropy (3) via a new parameter *q*.

#### 4.2.2. Rényi Entropy

Rényi entropy [11], which is a measure of entanglement in quantum information theory, is additive and preserves event independence. It is another important generalization of BG entropy, which is defined as follows:

(27) $$S_{R}=k_{B}\frac{\ln\sum_{i=1}^{n}{\left(p_{i}\right)}^{q}}{1-q}.$$

By assuming that all the states are equally probable, as in (2), it follows from (27) that

(28) $$S_{R}=k_{B}\ln n,$$

which is the same as BG entropy (1).

In fact, the Rényi entropy (27) can be written in terms of the Tsallis *q*-entropy by using the formal logarithm approach [52] as follows:

(29) $$\begin{array}{c} S_{R}=\frac{k_{B}}{1-q}\ln\left[1+\frac{1-q}{k_{B}}S_{q}\right]. \end{array}$$

A unique feature of Rényi entropy is that *it is additive*, which results from some more general Abé composition rule given by [49]:

(30) $$H\left(S_{A+B}\right)=H\left(S_{A}\right)+H\left(S_{B}\right)+\frac{\Upsilon }{k_{B}}H\left(S_{A}\right)H\left(S_{B}\right),$$

together with redefinition using the logarithm in the form

(31) $$L\left(S\right)=\frac{k_{B}}{\Upsilon }\ln\left(1+\frac{\Upsilon }{k_{B}}H\left(S\right)\right),$$

which applied to (29) gives an additive formula

(32) $$L\left(S_{A+B}\right)=L\left(S_{A}\right)+L\left(S_{B}\right),$$

where L(S) corresponds to the Rényi entropy, and H(S) corresponds to the Tsallis *q*-entropy. In such a formulation, one can write that the Rényi entropy fulfills the Abé rule (12), with the parameter Υ=0.

#### 4.2.3. Tsallis–Cirto δ-Entropy

The Tsallis–Cirto δ-entropy [4,9], sometimes also known in the literature as the Tsallis entropy, is yet another generalization of BG entropy (1) via the introduction of another nonextensivity parameter δ as follows:

(33) $$S_{\delta }=k_{B}\sum_{i=1}^{n}p_{i}{\left(\ln p_{i}\right)}^{\delta }\;\left(\delta >0,\delta \in R\right),$$

and this difference is easily recognized when one compares it with the Tsallis *q*-entropy (19) and with BG entropy (1).

Under the assumption that all the states are equally probable, as in (2), one determines from (33) that

(34) $$S_{\delta }=k_{B}{\left(\ln n\right)}^{\delta }\equiv k_{B}\ln^{\delta }n.$$

Making another assumption that we deal with two independent systems, *A* and *B*, fulfilling the condition $n^{A+B}=n^{A}\cdot n^{B}$, one realizes that the composition rule for the Tsallis–Cirto entropy (34) reads

(35) $${\left(\frac{S_{\delta ,A+B}}{k_{B}}\right)}^{1/\delta }={\left(\frac{S_{\delta ,A}}{k_{B}}\right)}^{1/\delta }+{\left(\frac{S_{\delta ,B}}{k_{B}}\right)}^{1/\delta },$$

which is another example of a composition rule, which is *different* from the Abé rule (12). We call this the *δ-addition rule* from now on. In fact, Tsallis and Cirto suggest that [4,9]

(36) $$\begin{array}{c} S_{\delta }=k_{B}{\left(\frac{S_{Bek}}{k_{B}}\right)}^{\delta }, \end{array}$$

where $S_{Bek}$ is the Bekenstein entropy (14). According to a new composition rule (35), one realizes that the Bekenstein entropy, as given by $S_{Bek}\propto {\left(S_{\delta }\right)}^{1/\delta }$, can be additive, while the Tsallis–Cirto entropy $S_{\delta }$ itself is nonadditive. Additionally, bearing in mind the definition of Bekenstein entropy for a Schwarzschild black hole (14), one can easily notice that for δ=3/2, the Tsallis–Cirto entropy (36) is proportional to the volume $S_{\delta }\propto M^{3}$, and so it is an extensive quantity in view of the standard definition of extensivity. We come back to this issue in Section 4.3.

#### 4.2.4. Tsallis q,δ-Entropy

The Tsallis q,δ-entropy generalizes both the Tsallis *q*-entropy (19) and the Tsallis-Cirto δ-entropy (33), combining them as follows [4,9]:

(37) $$S_{q,\delta }=k_{B}\sum_{i=1}^{n}p_{i}{\left(\ln_{q}p_{i}\right)}^{\delta }\;\left(\delta >0,q\in R,\delta \in R\right).$$

Now, both *q* and δ play the role of two independent nonextensivity parameters. By assuming that all the states are equally probable as in (2), one gets from (37) that

(38) $$S_{q,\delta }=k_{B}{\left(\ln_{q}n\right)}^{\delta }\equiv k_{B}\ln_{q}^{\delta }n.$$

The Tsallis q,δ-entropy does not fulfill the Abé addition rule or the δ-addition rule, though it fulfills the former in the limit δ→0 and the latter in the limit q→0.

#### 4.2.5. Tsallis–Jensen q,γ-Entropy

Recently, Tsallis and Jensen [38] proposed another generalization of BG entropy, which reads

(39) $$S_{q,\gamma }=k_{B}{\left[\frac{\ln\sum_{i=1}^{n}p_{i}^{q}}{1-q}\right]}^{\frac{1}{\gamma }}=k_{B}{\left(\frac{S_{R}}{k_{B}}\right)}^{\frac{1}{\gamma }},$$

where $S_{R}$ is the Rényi entropy and γ is a new parameter somewhat analogous to the parameter δ in the Tsallis–Cirto entropy (33).

Since the Rényi entropy possesses the BG limit for q→1, we can write [38]

(40) $$S_{1,\gamma }=k_{B}{\left(\frac{S_{BG}}{k_{B}}\right)}^{\frac{1}{\gamma }},$$

and analogously, if we take Bekenstein entropy (14) instead of the BG in (40), in the same limit q→1, we get

(41) $$S_{1,\gamma }^{Bek}=k_{B}{\left(\frac{S_{Bek}}{k_{B}}\right)}^{\frac{1}{\gamma }}.$$

Bearing in mind the additive composition rule (5) for the BG entropy, and using (40), one can write the additivity rule for $S_{1,\gamma }$ as follows:

(42) $${\left[S_{1,\gamma }\left(A+B\right)\right]}^{\gamma }={\left[S_{1,\gamma }\left(A\right)\right]}^{\gamma }+{\left[S_{1,\gamma }\left(B\right)\right]}^{\gamma }.$$

Similarly, taking into account the square root additivity rule (17) for the Bekenstein-like entropy (41), one can write the composition rule as follows:

(43) $${\left[S_{1,\gamma }^{Bek}\left(A+B\right)\right]}^{\gamma }={\left[S_{1,\gamma }^{Bek}\left(A\right)\right]}^{\gamma }+{\left[S_{1,\gamma }^{Bek}\left(B\right)\right]}^{\gamma }+2{\left[S_{1,\gamma }^{Bek}\left(A\right)S_{1,\gamma }^{Bek}\left(B\right)\right]}^{\frac{\gamma }{2}}.$$

Finally, since the Rényi entropy $S_{R}$ in (39) is in general additive according to the composition rule (32), then we can write a generic composition rule for the Tsallis–Jensen entropy (39) as follows:

(44) $${\left[S_{q,\gamma }\left(A+B\right)\right]}^{\gamma }={\left[S_{q,\gamma }\left(A\right)\right]}^{\gamma }+{\left[S_{q,\gamma }\left(B\right)\right]}^{\gamma },$$

and this is exactly the δ composition rule (35) with γ=1/δ.

Table 1 gives the summary of four different Tsallis invented entropies.

### 4.3. Barrow Fractal Horizon Δ-Entropy and Its Relation to Bekenstein and Tsallis–Cirto δ-Entropy

Barrow entropy [16] has no statistical roots at all. It is closely tied to black hole horizon geometry influenced by quantum fluctuations, which make an initially smooth black hole horizon a fractal composed of spheres, forming the so-called sphereflake. This structure is characterized by the fractal dimension $d_{f}$, which in extreme cases are either the surface or the volume, i.e., $2\leq d_{f}\leq 3$, resulting in an effective horizon area of $r^{d_{f}}$, where *r* is the black hole horizon radius. After quantum-motivated modification of the area, the entropy reads as follows:

(45) $$\begin{array}{c} S_{Bar}=k_{B}{\left(\frac{A}{A_{p}}\right)}^{1+\frac{\Delta }{2}}=k_{B}{\left(\frac{S_{Bek}}{k_{B}}\right)}^{1+\frac{\Delta }{2}}, \end{array}$$

where $S_{Bek}$ is Bekenstein entropy, *A* is the horizon area, $A_{p}$ is the Planck area, $A_{p}\propto l_{p}^{2}$ with $l_{p}$ is the Planck length, and Δ is the parameter related to the fractal dimension by the relation $\Delta =d_{f}-2$. In fact, 0≤Δ≤1 with the Δ→1 limit, yielding a maximally fractal structure, where the horizon area behaves effectively like a three-dimensional volume, and with the Δ→0 limit yielding the Bekenstein area law, where no fractalization occurs. Although Barrow entropy has geometrical roots and is not motivated by thermodynamics, it has the same form as Tsallis–Cirto δ entropy (36) [53], being also related to Bekenstein entropy $S_{Bek}$, as in (14), provided that

(46) $$\begin{array}{c} \delta =1+\frac{\Delta }{2}. \end{array}$$

However, the ranges of parameters δ and Δ are different—δ has only the bound δ>0, while 0≤Δ≤1 is equivalent to 1≤δ≤3/2. Thus, qualitatively, both entropic forms yield the same temperatures as a function of a black hole mass. Both the Tsallis–Cirto entropy limit δ→3/2 and the Barrow limit Δ→1 yield an extensive but still nonadditive entropy for black holes. In fact, the cosmological studies of Barrow entropy as holographic dark energy have been performed intensively [17,18,19,20,21,22,23,24,25,26,27,28,29,30,31,32,33,34], pointing towards this extensive case [18,38,54].

### 4.4. Landsberg U-Entropy

The Landsberg *U*-entropy is defined in relation to Tsallis *q*-entropy (25) as follows [39]:

(47) $$S_{U}=\frac{k_{B}}{1-q}\left(1-\frac{1}{\sum_{i=1}^{n}{\left(p_{i}\right)}^{q}}\right)=k_{B}\frac{1-\sum_{i=1}^{n}{\left(p_{i}\right)}^{q}}{q-1}\frac{1}{{\left(p_{i}\right)}^{q}}=\frac{S_{q}}{\sum_{i=1}^{n}{\left(p_{i}\right)}^{q}},$$

and it fulfills the Abé rule (12) for Υ=q−1 (cf. Appendix B). By assuming that all the states are equally probable as in (2), it simplifies (47) to the form

(48) $$S_{U}=n^{q-1}S_{q},$$

so it simply relates to Tsallis *q*-entropy.

### 4.5. Sharma–Mittal Entropy

The Sharma–Mittal (SM) entropy [12,55] combines the Rényi entropy with the Tsallis q−entropy, and it is defined as follows:

(49) $$\begin{array}{c} S_{SM}=\frac{k_{B}}{R}\left[{\left(\sum_{i=1}^{n}{\left(p_{i}\right)}^{q}\right)}^{\frac{R}{1-q}}-1\right], \end{array}$$

where *R* is another dimensionless parameter apart from *q*. For equally probable states in (2), one determines from (49) that [56]

(50) $$\begin{array}{c} S_{SM}=\frac{k_{B}}{R}\left\{{\left[1+\frac{1-q}{k_{B}}S_{q}\right]}^{\frac{R}{1-q}}-1\right\}, \end{array}$$

where the R→1−q limit yields the Tsallis entropy, and the R→0 limit yields the Rényi entropy. It is interesting to notice that the SM entropy obeys the composition rule of Abé (12) for Υ=1 (cf. Appendix C).

### 4.6. Kaniadakis Entropy

The Kaniadakis entropy [14,15,24] results from taking into account Lorentz transformations of special relativity. It is a single *K*-parameter (−1<K<1) deformation of BG entropy (1), with the *K* parameter related to the dimensionless rest energy of the various parts of a multibody relativistic system. The basic definition of Kaniadakis entropy, which directly generalizes BG entropy, reads as follows:

(51) $$S_{K}=-k_{B}\sum_{i=1}^{n}p_{i}\ln_{K}p_{i},$$

where $p_{i}$ is the probability distribution and *n* is the total number of states, as mentioned in Section 2. The formula (51) introduces the *K*-logarithm

(52) $$\ln_{K}x\equiv \frac{x^{K}-x^{-K}}{2K}=\frac{1}{K}\sinh\left(K\ln x\right)$$

with some basic properties like $\ln_{K}x^{-1}=-\ln_{K}x$ and $\ln_{-K}x=\ln_{K}x$, giving the standard logarithm lnx in the limit K→0. An equivalent definition of Kaniadakis entropy, which can be obtained after the application of the K−logarithm (52), reads as follows:

(53) $$S_{K}=-k_{B}\sum_{i=1}^{n}\frac{{\left(p_{i}\right)}^{1+K}-{\left(p_{i}\right)}^{1-K}}{2K}.$$

The K−deformed logarithm is associated with the K−exponential as follows:

(54) $$\exp_{K}x=\exp\left[\frac{1}{K}\mathrm{arcsinh}\left(Kx\right)\right]={\left(\sqrt{1+K^{2}x^{2}}+Kx\right)}^{1/K},$$

and it fulfills some basic relations like $\exp_{K}\left(x\right)\exp_{K}\left(-x\right)=1$, and $\exp_{K}\left(x\right)\exp_{-K}\left(x\right)$, giving the standard exponential function exp(x) in the limit K→0. In fact, the K−logarithm and K−exponential are the inverse functions, which means that they fulfill the relation

(55) $$\ln_{K}\left(\exp_{K}x\right)=\exp_{K}\left(\ln_{K}x\right)=x.$$

The K−logarithm fulfills a generalized composition rule, which reads as follows:

(56) $$\ln_{K}\left(xy\right)=\ln_{K}x\sqrt{1+K^{2}{\left(\ln_{K}y\right)}^{2}}+\ln_{K}y\sqrt{1+K^{2}{\left(\ln_{K}x\right)}^{2}},$$

and it admits the standard logarithm rule ln(xy)=lnx+lny in the limit K→0. The rule (56) comes from the definition of the K−sum:

(57) $${\left(x⊕y\right)}_{K}=x\sqrt{1+K^{2}y^{2}}+y\sqrt{1+K^{2}x^{2}},$$

where one replaced x→lnx and y→lny, giving the standard additivity rule ${\left(x⊕y\right)}_{K}=x+y$ in the limit K→0. Using the definition of Kaniadakis entropy (51), as well as the K−logarithm addition rule, we can write down the Kaniadakis entropy composition rule as follows:

(58) $$S_{K}\left(A+B\right)=S_{K}\left(A\right)\sqrt{1+\frac{K^{2}}{k_{B}^{2}}S_{K}\left(B\right)}+S_{K}\left(B\right)\sqrt{1+\frac{K^{2}}{k_{B}^{2}}S_{K}\left(A\right)},$$

which we start calling the *K−addition rule* from now on. It is interesting to note that through the application of the K−sum [14], defined as

(59) $${\left(x⊗y\right)}_{K}=\frac{1}{K}\sinh\left[\frac{1}{K}\mathrm{arcsinh}\left(Kx\right)\mathrm{arcsinh}\left(Ky\right)\right],$$

one has for the K−logarithm

(60) $$\ln_{K}\left[{\left(x⊗y\right)}_{K}\right]=\ln_{K}x+\ln_{K}y,$$

so that applying it to (51), the Kaniadakis entropy (in full analogy to the q−product of Tsallis given by (22)), can take a completely *additive form* as below (cf. the definition of additivity (4) for statistically independent systems)

(61) $$S_{K}{\left(A+B\right)}_{K}=S_{K}\left(p_{A}p_{B}\right)=S_{K}\left(p_{A}\right)+S_{K}\left(p_{B}\right)=S_{K}\left(A\right)+S_{K}\left(B\right).$$

Using the K−product, the Kaniadakis entropy can also be *extensive*

(62) $$S_{K}\left(x^{⊗r}\right)=rS_{K}\left(x\right),$$

where r= const., and it comes as a result of the identity

(63) $$\ln_{K}\left(x^{⊗r}\right)=r\ln_{rK}\left(x\right).$$

Finally, in analogy to the previous considerations, and under the assumption that all the states are equally probable, as in (2), one gets from (51) that

(64) $$\ln_{K}p_{i}=-\frac{1}{K}\frac{e^{K\ln n}-e^{-K\ln n}}{2},$$

which can further be transformed into

(65) $$S_{K}=\frac{k_{B}}{K}\sinh\left(\frac{K}{k_{B}}S\right),$$

where $S=k_{B}\ln n$ is the BG entropy (3).

### 4.7. Thermal Equilibrium Temperature vs. Equilibrium Temperature for Nonextensive Entropies

One important issue related to nonextensive systems is the formulation of a proper definition of the thermal equilibrium temperature, which is necessary according to the zeroth law of thermodynamics [36,52,57]. This problem can be solved by using the notion of the *effective equilibrium temperature* based on the equilibrium condition [46], recently discussed in more detail in ref. [42]. In defining this temperature, one uses an analogy to some strongly coupled quantum systems which can be in equilibrium at the effective temperature, but not in the thermal equilibrium, as for the extensive systems. The equilibrium temperature is obtained by maximizing the composition rule (9) with the fixed total energy of the system $U_{AB}=U_{A}+U_{B}$, which leads to the conditions $\delta S_{AB}=0$, with $S_{AB}=g\left(S_{A},S_{B}\right)$, and δU(A+B)=0. The equilibrium temperature $T_{eq}$ then reads

(66) $$T_{eq}\equiv \frac{1}{k_{B}\beta^{*}}=\frac{\frac{\partial g}{\partial S_{B}}}{k_{B}\beta_{A}}=\frac{\frac{\partial g}{\partial S_{A}}}{k_{B}\beta_{B}},$$

where we have defined β for each system, i.e.,

(67) $$k_{B}\beta_{A}=\frac{\partial S_{A}}{\partial U_{A}}\;\mathrm{and}\;k_{B}\beta_{B}=\frac{\partial S_{B}}{\partial U_{B}}.$$

The application of the above procedure to the specific system described by the Tsallis *q*-entropy (19), which fulfills the Abé composition rule (12) as an example of the general composition rule (9), gives the equilibrium temperature [42]

(68) $$T_{eq}=T_{R}=\left(1+\frac{1-q}{k_{B}}S_{q}\right)\frac{1}{k_{B}\beta },$$

which is the Rényi temperature $T_{R}$ [58] corresponding to Rényi entropy $S_{R}$ given by (29) according to the Clausius formula (16).

It is interesting that the Rényi entropy and the Rényi effective equilibrium temperature can be defined on a horizon of a Schwarzschild black hole [5,6,7,8,59] by assuming that the Bekenstein entropy $S_{Bek}$ given by (14) is the Tsallis entropy $S_{q}$ in (29) and (68).

A similar procedure of introducing the equilibrium temperature can be performed for the Tsallis–Cirto δ-entropy by calculating the corresponding temperature from the Clausius relation (16) as follows: [42,58]

(69) $$T_{\delta }=\frac{T_{H}}{\delta }{\left(\frac{S_{Bek}}{k_{B}}\right)}^{1-\delta },$$

which scales with $1/M^{2}$ for δ=3/2, i.e., $T_{\delta }\propto 1/M^{2}$ for a Schwarzschild black hole.

### 4.8. Classification of Entropies

Bearing in mind all the considerations of Section 4, we present a summary of the additivity and extensivity properties of the entropies in the Table 2.

There is the whole group of Tsallis-invented thermodynamical entropies, which generalize BG entropy in some different ways (cf. also Table 1). They obey either the Abé composition rule or the δ-addition rule. The Tsallis *q*-entropy relates to both the Rényi and the Landsberg U entropies, while it is generalized by the Sharma–Mittal entropy. On the other hand, the Tsallis–Cirto δ-entropy is related to the Barrow entropy and the Tsallis–Jensen q,γ entropy and, interestingly, it points towards extensivity, when observations are taken into account [18,38,54]. The Kaniadakis entropy seem to form a separate group of nonextensive entropies because of its hyperbolic formulation as a consequence of relativity theory being taken into account, but it still has a BG limit. In fact, all the entropies in our study have a BG limit, except Bekenstein entropy, which is not formulated as proper statistics. However, it is composable, though its composition rule is unique among any other rules, which are often shared with themselves. Additionally, in the microscopic counting of states in the string theory [60,61], the nonextensive Bekenstein formula is recovered, though it was also found that some higher-dimensional extremal black holes allow for Bekenstein entropy, which is consistent with the Boltzmann–Gibbs extensive limit.

### 4.9. Generalized Four- and Five-Parameter Entropic Forms

There exists a four-parameter entropic formula [62], which reads as follows:

(70) $$S_{g}\left(\alpha_{\pm },\beta ,\sigma \right)=\frac{k_{B}}{\sigma }\left[{\left(1+\frac{\alpha_{+}}{\beta }\frac{S}{k_{B}}\right)}^{\beta }-{\left(1+\frac{\alpha_{-}}{\beta }\frac{S}{k_{B}}\right)}^{-\beta }\right],$$

as well as the five-parameter formula [63] of the following form:

(71) $$S_{g}\left(\alpha_{\pm },\beta ,\sigma ,\epsilon \right)=\frac{k_{B}}{\sigma }\left\{{\left[1+\frac{1}{\epsilon }\tanh\left(\frac{\epsilon \alpha_{+}}{\beta }\frac{S}{k_{B}}\right)\right]}^{\beta }-{\left[1+\frac{1}{\epsilon }\tanh\left(\frac{\epsilon \alpha_{-}}{\beta }\frac{S}{k_{B}}\right)\right]}^{-\beta }\right\},$$

Both these formulas generalize some of the entropies which are contained in Table 2 and have the following limits:

1. if ϵ→0, then one recovers the Tsallis–Cirto (33) and Barrow (45) entropies;
2. if ϵ→0, $\alpha_{-}\to 0$, β→0, and $\alpha_{+}/\beta$ is finite, then one recovers the Rényi entropy (29);
3. if ϵ→0, $\alpha_{-}\to 0$, $\sigma =\alpha_{+}=R$, and β=R/δ, then one recovers the Sharma–Mittal entropy Formula (50), though only when one replaces Tsallis *q*-entropy $S_{q}$ with Tsallis–Cirto δ-entropy $S_{\delta }$;
4. if ϵ→0, β→∞, $\alpha_{+}=\alpha_{-}=\sigma /2=K$, then one recovers Kaniadakis entropy (65).

These entropies have an important advantage for cosmology. Namely, they are singular-free at the cosmological bounce, which is characterized by vanishing of the Hubble parameter H=0 in bouncing scenarios [64]. Additionally, they allow for microscopic interpretation [65,66].

Since all of them are in general nonadditive and nonextensive, it is not necessary to discuss them in more detail, though they may recover BG behavior in some special cases. However, we will not consider these cases in detail here.

## 5. Summary and Discussion

Beginning with the underlying properties of the Boltzmann–Gibbs classical entropy, we have investigated the problem of nonextensity and nonadditivity in thermodynamics, aiming towards gravitational systems which admit long-range interactions. The focus has been on such extensions of Boltzmann–Gibbs entropic form, which allow for various deformations of it via some new parameters modifying the space of microstates Ω. These new parameters are given as some interpretations according to a deformation and can be enumerated as the Tsallis nonextensivity q−parameter; the Tsallis–Cirto nonextensivity δ−parameter, which is equivalent to the Barrow fractality Δ−parameter; the Tsallis–Jensen nonextensivity parameter γ; the Sharma–Mittal R−parameter; and the Kaniadakis relativistic K−deformation parameter.

The entropies under study may fulfill some composition rules , including the Abé rule and δ−addition rule, both of which are nonaddtitive. Bekenstein entropy obeys some other nonadditive rule, called the square root rule, which is somewhat similar, but not the type of the Abé rule. The Kaniadakis entropy can be made additive within a special K−deformed algebra, and it reaches the standard Boltzmann–Gibbs additive rule in the limit K→0. The same may refer to other entropies which are composable, such as the Tsallis *q*-entropy and the Bekenstein entropy. A separate matter is whether these “made-additive” entropies have a proper physical interpretation. Another point that we have discussed was the definition of the equilibrium temperature for nonextensive systems, in contrast to the thermal equilibrium temperature, which is ambiguous for these systems.

We have presented two important comparative tables with the additivity, extensivity, and composability properties of the entropies under study, and the relations between them have been shown explicitly. To our knowledge, such a collective comparison of entropic forms have not yet been presented in the literature. In view of the recent interest of both astrophysicists and cosmologists in the application of the plethora of alternatives to Boltzmann–Gibbs entropies, this paper may then serve as a useful guide to these applications. It is worth noting that a number of these entropies are considered suitable as the candidates for holographic dark energy; therefore, the presented summary gives some structure to their general properties in a systematic way, which can help astrophysicists and cosmologists to sort them out into categories, which can then be systematically verified by the data. Currently, it seems that most of the tests are being performed mainly for some particular types of entropies without significant regard to their relations to others.

## Figures and Tables

**Table 1:** Tsallis entropies.

Entropy Type	Extensivity	Additivity	Abé Addition Rule	δ-Addition Rule
Boltzmann–Gibbs $S_{BG}$	yes	yes	yes, Υ=0	yes, δ=1
Tsallis $S_{q,1}=S_{q}$	no	no	yes, Υ=1−q	no
Tsallis–Cirto $S_{1,\delta }=S_{\delta }$	no	no	no	yes
General Tsallis $S_{q,\delta }$	no	no	no	no
Tsallis–Jensen $S_{q,\gamma }$	no	no	no	yes, δ=1/γ

**Table 2:** The additivity, extensivity, and composability properties of entropies.

Entropy Type	Extensivity	Additivity	Abé Rule	δ-Rule	*K*-Rule
Boltzmann–Gibbs $S_{BG}$	yes	yes	yes, Υ=0	yes, δ=1	yes, K=0
Bekenstein $S_{Bek}$	no	no *	no	no	no
Tsallis *q*-entropy $S_{q}$	no	no	yes, Υ=1−q	no	no
Tsallis–Cirto $S_{\delta }$ $\left(\delta \neq \frac{3}{2}\right)$	no	no	no	yes	no
Tsallis–Cirto $S_{\delta }$ $\left(\delta =\frac{3}{2}\right)$	yes	no	no	yes, $\delta =\frac{3}{2}$	no
Barrow $S_{Bar}=S_{Bek}$ (Δ=0)	no	no *	no	no	no
Barrow $S_{Bar}$ (0<Δ<1)	no	no	no	yes	no
Barrow $S_{Bar}$ (Δ=1)	yes	no	no	yes, $\delta =\frac{3}{2}$	no
Rényi $S_{R}$	no	yes	yes, Υ=0	no	no
Landsberg *U*-entropy $S_{U}$	no	no	yes, Υ=q−1	no	no
Kaniadakis $S_{K}$	no	no	no	no	yes
Sharma–Mittal $S_{SM}\left(q,R\right)$	no	no	yes, Υ=R	no	no
Tsallis q,δ-entropy $S_{q,\delta }$	no	no	no	no	no
Tsallis–Jensen $S_{q,\gamma }$	no	no	no	yes, δ=1/γ	no
Tsallis–Jensen $S_{1,\gamma }$	no	no	no	yes, δ=1/γ	no
Tsallis–Jensen $S_{q,1}=S_{R}$	no	yes	yes, Υ=0	no	no

* obeys square root composition rule (17).

## Data Availability

No data was generated.

## Appendix A. Equivalent Forms of Tsallis q-Entropy

There are three equivalent forms of Tsallis q−entropy given by (19), which we can label according to their appearance in (19): $S_{qI},S_{qII},S_{qIII}$. We show their equivalence by reducing each of them to the form (25) via the application the probability sum rule $\sum p_{i}=1$ and Formula (20). For $S_{qI}$, one has

(A1) $$\begin{array}{c} S_{qI}\equiv k_{B}\sum_{i=1}^{n}p_{i}\ln_{q}\frac{1}{p_{i}}=k_{B}\sum_{i=1}^{n}p_{i}\frac{{\left(\frac{1}{p_{i}}\right)}^{1-q}-1}{1-q}=k_{B}\sum_{i=1}^{n}\frac{{\left(p_{i}\right)}^{q}-1}{1-q}=k_{B}\frac{1-\sum_{i=1}^{n}{\left(p_{i}\right)}^{q}}{q-1}, \end{array}$$

which is equivalent to (25). For $S_{qII}$, one has

(A2) $$\begin{array}{c} S_{qII}=-k_{B}\sum_{i=1}^{n}{\left(p_{i}\right)}^{q}\ln_{q}p_{i}=-k_{B}\sum_{i=1}^{n}{\left(p_{i}\right)}^{q}\frac{{\left(p_{i}\right)}^{1-q}-1}{1-q}=-k_{B}\frac{\sum_{i=1}^{n}\left[\left(p_{i}\right)-{\left(p_{i}\right)}^{q}\right]}{1-q}=k_{B}\frac{1-\sum_{i=1}^{n}{\left(p_{i}\right)}^{q}}{q-1}, \end{array}$$

which is equivalent to (25). For $S_{qIII}$, one has

(A3) $$\begin{array}{c} S_{qIII}=-k_{B}\sum_{i=1}^{n}\ln_{2-q}p_{i}=-k_{B}\sum_{i=1}^{n}\left(p_{i}\right)\frac{{\left(p_{i}\right)}^{q-1}-1}{q-1}=-k_{B}\sum_{i=1}^{n}\frac{{\left(p_{i}\right)}^{q}-\left(p_{i}\right)}{q-1}=k_{B}\frac{1-\sum_{i=1}^{n}{\left(p_{i}\right)}^{q}}{q-1}, \end{array}$$

where we have applied a redefined q−logarithm (20) with $q^{\prime }=2-q$. This finishes the proof.

## Appendix B. Validity of Abé Composition Rule for Tsallis *q*−Entropy and Landsberg *U*−Entropy

We first assume two probabilistically independent systems, *A* and *B*, fulfilling

(A4) $$\sum_{i=1}^{n}\sum_{j=1}^{m}p_{ij}^{A+B}=\sum_{i=1}^{n}\sum_{j=1}^{m}p_{i}^{A}p_{j}^{B}$$

for every i,j, which gives

(A5) $$\begin{array}{c} \sum_{i=1}^{n}\sum_{j=1}^{m}{\left(p_{ij}^{A+B}\right)}^{q}=\sum_{i=1}^{n}\sum_{j=1}^{m}{\left(p_{i}^{A}p_{j}^{B}\right)}^{q}=\sum_{i=1}^{n}\sum_{j=1}^{m}{\left(p_{i}^{A}\right)}^{q}{\left(p_{j}^{B}\right)}^{q}. \end{array}$$

Following the definition of Tsallis q−entropy (25), one can write it using (A5):

(A6) $$\begin{array}{c} S_{q}\left(A+B\right)=k_{B}\frac{1-\sum_{i=1}^{n}\sum_{j=1}^{m}{\left(p_{ij}^{A+B}\right)}^{q}}{q-1}=k_{B}\frac{1-\sum_{i=1}^{n}\sum_{j=1}^{m}{\left(p_{i}^{A}\right)}^{q}{\left(p_{j}^{B}\right)}^{q}}{q-1}, \end{array}$$

as well as

(A7) $$S_{q}\left(A\right)=k_{B}\frac{1-\sum_{i=1}^{n}{\left(p_{i}^{A}\right)}^{q}}{q-1},$$

and analogously for $S_{q}\left(B\right)$.

Following the Abé rule (12) we can then write the right-hand side of it as follows:

(A8) $$\begin{array}{ccc} & & RHS=S_{q}\left(A\right)+S_{q}\left(B\right)+\frac{\Upsilon }{k_{B}}S_{q}\left(A\right)S_{q}\left(B\right)=k_{B}\frac{1-\sum_{i=1}^{n}{\left(p_{i}^{A}\right)}^{q}}{q-1}+k_{B}\frac{1-\sum_{j=1}^{m}{\left(p_{j}^{A}\right)}^{q}}{q-1}\\ & & +\Upsilon k_{B}\left(\frac{1-\sum_{i=1}^{n}{\left(p_{i}^{A}\right)}^{q}}{q-1}\right)\left(\frac{1-\sum_{j=1}^{m}{\left(p_{i}^{A}\right)}^{q}}{q-1}\right)=\frac{k_{B}}{q-1}\left[1-\sum_{i=1}^{n}{\left(p_{i}^{A}\right)}^{q}+1-\sum_{i=1}^{n}{\left(p_{j}^{B}\right)}^{q}\right.\\ & & \left.+\frac{Y}{q-1}-\frac{Y}{q-1}\sum_{i=1}^{n}{\left(p_{i}^{A}\right)}^{q}-\frac{Y}{q-1}\sum_{i=1}^{n}{\left(p_{j}^{B}\right)}^{q}+\frac{Y}{q-1}\sum_{i=1}^{n}{\left(p_{i}^{A}\right)}^{q}\sum_{j=1}^{m}{\left(p_{j}^{B}\right)}^{q}\right], \end{array}$$

which after selecting Υ=1−q, cancels six out of eight terms giving on the base of (A5) that

(A9) $$\begin{array}{c} RHS=k_{B}\frac{1-\sum_{i=1}^{n}\sum_{j=1}^{m}{\left(p_{i}^{A}\right)}^{q}{\left(p_{j}^{B}\right)}^{q}}{q-1}=k_{B}\frac{1-\sum_{i=1}^{n}\sum_{j=1}^{m}{\left(p_{ij}^{A+B}\right)}^{q}}{q-1}=S_{q}\left(A+B\right). \end{array}$$

The proof of applicability of the Abé rule for Landsberg U−entropy (47) proceeds analogously, provided that Υ=q−1 instead.

## Appendix C. Validity of Abé Composition Rule for Sharma–Mittal Entropy

Let us write the Abé rule for the Sharma–Mittal entropy as follows:

(A10) $$S_{SM}\left(A+B\right)=S_{SM}\left(A\right)+S_{SM}\left(B\right)+\frac{\Upsilon }{k_{B}}S_{SM}\left(A\right)S_{SM}\left(B\right)$$

with

(A11) $$S_{SM}\left(A+B\right)=\frac{k_{B}}{R}\left[{\left(\sum_{i=1}^{n}\sum_{j=1}^{m}{\left(p_{ij}^{A+B}\right)}^{q}\right)}^{\frac{R}{1-q}}-1\right]$$

and

(A12) $$S_{SM}\left(A\right)=\frac{k_{B}}{R}\left[{\left(\sum_{i=1}^{n}{\left(p_{i}^{A}\right)}^{q}\right)}^{\frac{R}{1-q}}-1\right].$$

Calculation of the RHS of (A10) gives the following:

(A13) $$\begin{array}{ccc} & & RHS=\frac{k_{B}}{R}\left[{\left(\sum_{i=1}^{n}{\left(p_{i}^{A}\right)}^{q}\right)}^{\frac{R}{1-q}}-1\right]+\frac{k_{B}}{R}\left[{\left(\sum_{j=1}^{m}{\left(p_{j}^{B}\right)}^{q}\right)}^{\frac{R}{1-q}}-1\right]+\frac{\Upsilon }{k_{B}}\frac{k_{B}}{R}\left[{\left(\sum_{i=1}^{n}{\left(p_{i}^{A}\right)}^{q}\right)}^{\frac{R}{1-q}}-1\right]\times \\ & & \frac{k_{B}}{R}\left[{\left(\sum_{j=1}^{m}{\left(p_{j}^{B}\right)}^{q}\right)}^{\frac{R}{1-q}}-1\right]=\frac{k_{B}}{R}\left[{\left(\sum_{i=1}^{n}{\left(p_{i}^{A}\right)}^{q}\right)}^{\frac{R}{1-q}}-1+{\left(\sum_{j=1}^{m}{\left(p_{j}^{B}\right)}^{q}\right)}^{\frac{R}{1-q}}-1\right.\\ & & \left.+\frac{\Upsilon }{R}{\left(\sum_{i=1}^{n}{\left(p_{i}^{A}\right)}^{q}\right)}^{\frac{R}{1-q}}{\left(\sum_{j=1}^{m}{\left(p_{j}^{B}\right)}^{q}\right)}^{\frac{R}{1-q}}-\frac{\Upsilon }{R}{\left(\sum_{i=1}^{n}{\left(p_{i}^{A}\right)}^{q}\right)}^{\frac{R}{1-q}}-\frac{\Upsilon }{R}{\left(\sum_{j=1}^{m}{\left(p_{j}^{B}\right)}^{q}\right)}^{\frac{R}{1-q}}+\frac{\Upsilon }{R}\right]\\ & & =\frac{k_{B}}{R}\left[{\left(\sum_{i=1}^{n}\sum_{j=1}^{m}{\left(p_{i}^{A}\right)}^{q}{\left(p_{j}^{B}\right)}^{q}\right)}^{\frac{R}{1-q}}-1\right]=\frac{k_{B}}{R}\left[{\left(\sum_{i=1}^{n}\sum_{j=1}^{m}{\left(p_{ij}^{A+B}\right)}^{q}\right)}^{\frac{R}{1-q}}-1\right]=LHS, \end{array}$$

where we have taken Υ=R, and applied (A5).
